# Simvastatin-Induced Ferroptosis in Orbital Fibroblasts in Graves’ Ophthalmopathy

**DOI:** 10.1167/iovs.66.1.56

**Published:** 2025-01-24

**Authors:** Lujue Wang, Yuan Li, Tongxin Niu, Jing Deng, Yuxian Shi, Yating Liu, Boding Tong, Xin Qi, Dan Cao, Yongguang Tao, Yunping Li

**Affiliations:** 1 Department of Ophthalmology, The Second Xiangya Hospital of Central South University, Changsha, Hunan, China; 2 Hunan Clinical Research Center of Ophthalmic Disease, Changsha, Hunan, China; 3 National Clinical Research Center for Metabolic Diseases, The Second Xiangya Hospital of Central South University, Changsha, Hunan, China; 4 Guizhou Provincial People's Hospital, Guiyang, China; 5 Cancer Research Institute, School of Basic Medicine, and Key Laboratory of Carcinogenesis and Cancer Invasion, Ministry of Education, Central South University, Changsha, China

**Keywords:** graves’ ophthalmopathy, simvastatin, ferroptosis

## Abstract

**Purpose:**

Graves’ ophthalmopathy (GO), the most common extrathyroidal manifestation of Graves’ disease, is disabling and disfiguring. Recent studies have shown that statins have a protective effect on individuals with GO. Statins were reported to trigger ferroptosis in some disorders, but little is known about whether statins protect against GO via ferroptosis. The aim of this study was to explore whether ferroptosis is involved in the protective effect of simvastatin on GO.

**Methods:**

GO-OFs, which are orbital fibroblasts (OFs) derived from individuals with GO, were analyzed for lipogenesis by RT-qPCR and Red Oil O staining posttreatment with simvastatin. CCK-8 assays, flow cytometric analysis, and transmission electron microscopy (TEM) were used to compare the sensitivity of GO-OFs and control-OFs to erastin-induced ferroptosis. The ferroptosis levels in the GO-OFs were evaluated by measuring cell viability, reactive oxygen species (ROS) levels, and lipid peroxidation levels and performing TEM analysis after treatment with simvastatin and Fer-1.

**Results:**

The GO-OFs were resistant to erastin-induced ferroptosis. The viability and lipogenesis of the GO-OFs were significantly decreased, while the levels of ROS, lipid peroxidation, and the ferroptosis marker ACLS4 were increased upon treatment with simvastatin.

**Conclusions:**

Our study indicated that ferroptosis plays an important role in the pathogenesis of GO and that simvastatin may induce ferroptosis, suggesting that this drug could serve as a novel therapeutic agent for GO.

Graves’ ophthalmopathy (GO), also known as thyroid-associated ophthalmopathy, is an organ-specific autoimmune disease that negatively impacts individuals’ appearance, psychosocial well-being, and social functions; exacerbates visual impairment in 3% to 5% of cases; and results in major burden on individuals and society.^1^ GO is characterized by orbital inflammatory infiltration and activation of orbital fibroblasts (OFs), which mediate de novo adipogenesis, excessive production of hyaluronan, myofibroblast differentiation, and ultimately tissue fibrosis. The pathogenesis of GO is still unclear. For active GO, the effective rate of the mainstream therapy intravenous glucocorticoids (ivGCs) is approximately 60%, and this treatment results in various side effects, such as hyperglycemia and osteoporosis.^2^ Mild inactive GO benefits little from medical treatments and tends to be remedied with surgery due to persistent exophthalmos and cosmetic concerns. The discovery of more effective and economical treatments for GO is a critical unmet need.

Statins (such as simvastatin) are a class of drugs used to lower blood cholesterol levels through the inhibition of 3-hydroxy-3-methylglutaryl-CoA reductase (HMGCR), a rate-limiting enzyme in the mevalonate pathway, which mediates the synthesis of cholesterol. Recently, statins have been shown to dramatically slow disease development in individuals with GO.^3^ The addition of statins to an ivGC regimen can improve GO outcomes, suggesting that statins might increase the benefits of ivGCs and reduce the dose, thereby minimizing side effects.^4^ Since both oxidative stress and the release of proinflammatory cytokines are involved in GO, hypercholesterolemia is known to exert systemic proinflammatory effects, and the anti-inflammatory and immunomodulatory effects of statins may explain this effect.^5^^,^^6^ However, nonstatin lipid-lowering medications did not affect the development of GO in individuals with Graves’ disease, and there was no difference in low-density lipoprotein cholesterol between GO responders and nonresponders receiving statin treatment.^4^^,^^7^^,^^8^ The precise molecular mechanism underlying the effect of statins on GO has not been fully elucidated. Some studies have reported that simvastatin can inhibit the TGF-β–induced differentiation of GO-derived fibroblasts into myofibroblasts^9^ and inhibit the expression of the proliferation and differentiation gene *CYR61*.^10^ Statins have been shown to suppress early and late lipogenesis-related gene expression in 3T3-L1 preadipocytes and OFs^11^ and promote ferroptosis-related senescence in adipose tissue.^12^ Therefore, the effect of statins on GO might be related to their pleiotropic actions rather than to lowering cholesterol levels. Current evidence shows that statins can promote apoptosis, autophagy, the unfolded protein response,^13^ and ferroptosis by blocking the mevalonate pathway.^14^^–^^16^ In particular, statins can inhibit the biosynthesis of glutathione peroxidase 4 (GPX4) and coenzyme Q10 (CoQ10) and thus promote ferroptosis by decreasing the generation of isopentenyl pyrophosphate in the mevalonate pathway.^17^^,^^18^ This finding indicates that ferroptosis may be involved in the benefits of statins in individuals with GO.

Ferroptosis is a type of regulated cell death that is essential for the maintenance and development of homoeostasis in mammals.^19^ Lipid peroxidation and iron metabolism are the key regulatory processes in ferroptosis.^20^^,^^21^ Emerging evidence suggests a direct link between ferroptosis and mitochondrial dysfunction, with the excessive production of reactive oxygen species (ROS) due to uncontrolled mitochondrial respiration being considered a key trigger.^22^ Beyond these fundamental processes, various biological mechanisms can modulate the initiation and surveillance of ferroptosis, including the regulation of glutathione synthesis, the function of iron chelators, the expression of ferritin, and the equilibrium between pro-oxidant and antioxidant systems.^23^ These intricate regulatory processes collectively influence the progression of ferroptosis. A recent study revealed that OFs derived from GO (GO-OFs) are more resistant to ferroptosis than normal control OFs when cystine is depleted and/or ferroptosis inducers (erastin) are used.^24^ This finding suggested that ferroptosis may also be involved in the pathophysiology of GO. Therefore, ferroptosis resistance may be a potential therapeutic target in GO treatment.^24^^,^^25^

Despite the ability of statins to improve the outcome of individuals with GO, the direct effects of statins on ferroptosis resistance in GO-OFs have not been well studied. Therefore, we investigated the effects of simvastatin on the death of GO-OFs, focusing on ferroptosis. These results may provide a new direction for GO therapy.

## Materials and Methods

### Adipose Tissue Collection and Ethics Statements

Orbital adipose tissue samples were collected from 10 individuals diagnosed with GO and from 5 subjects without GO during surgery at the ophthalmology department of the Second Xiangya Hospital, Central South University, China; the results are listed in Tables 1 and 2, respectively. There was no notable difference in age or sex between the GO and control groups. All the included subjects were euthyroid for at least 3 months before surgery, and none were taking statins. The exclusion criteria were as follows: individuals with other systemic autoimmune or inflammatory diseases and individuals with diabetes mellitus. The acquisition of all tissue specimens was conducted in compliance with the guidelines outlined in the Declaration of Helsinki, with the approval of the Institutional Human Research Ethics Committee of the Second Xiangya Hospital, Central South University. Written informed consent was obtained from each individual.

**Table 1.** Clinical Characteristics of Individuals With GO in the Study

Characteristics	GO
Age, mean/range, y	40.4/22–67
Gender, male/female, *n*	8/2
Smoking, yes/no, *n*	2/8
GD history, yes/no, *n*	10/0
Treatment for GD, *n*	
Methimazole	9
Radioactive iodine therapy^*^	2
Thyroid surgery	0
None	1
Duration of GO, range (mean ± SD), mo	4–17 (6.9 ± 4.7)
CAS score (≤3), *n*	
0	5
1	2
2	3
3	0
Proptosis, range, right/left, mm	18–23/17–25
Previous treatment, *n*	
Radiation	0
GCs	5
None	5

* Two (out of 10) individuals were treated with methimazole as well as radioiodine therapy. CAS, Clinical Activity Score; GC, glucocorticoid; GD, Graves’ disease.

**Table 2.** Clinical Characteristics of Individuals Without GO in the Study

Characteristics	Non-GO
Age, mean/range, y	31.4/19-59 years
Gender, male/female, *n*	4/1
Smoking, yes/no, *n*	0/5
GD history, yes/no, *n*	0/5
GO history, yes/no, *n*	0/5
Diagnosis, *n*	
Ptosis	1
Orbital fat prolapse	1
Entropion and trichiasis	2
Orbital cavernous hemangioma	1

GD, Graves’ disease.

### Cell Culture

Orbital adipose tissues obtained from surgery were cut into pieces approximately 1 × 1 mm after removal of blood vessels and then promptly washed in PBS (Gibco, Anaheim, California, USA) supplemented with 1% penicillin/streptomycin (Gibco). Next, the tissues were rinsed twice with PBS, chopped with scissors, and digested at 37°C with 1 mg/mL collagenase type I (BioFroxx, Pfungstadt, Germany). After 30 to 60 minutes of digestion, Dulbecco's modified Eagle's medium (DMEM; Gibco) supplemented with 20% fetal bovine serum (FBS; Gibco, USA) was added. Following centrifugation, the OFs in the precipitate were cultured in a culture flask with proliferation media (DMEM-basic supplemented with 10% FBS) at 37°C in a 5% CO_2_ humidified incubator. When the OFs reached 80% to 90% confluence, they were digested and passaged using 0.25% ethylenediamine tetraacetate-free trypsin (Gibco). OFs between the second and eighth passages were used for the subsequent experiments. Each part of the following experiment was repeated for at least three consecutive and independent specimens. OFs from both sexes were employed without regard for sex.

### Immunofluorescence Assay

OFs were cultured in a monolayer, fixed for 15 minutes with 4% paraformaldehyde (Beyotime, Shanghai, China), and then rinsed in cold PBS. Following permeabilization for 15 minutes with PBS/0.1% Triton X-100 (Servicebio, Wuhan, China), blocking for 1 hour at room temperature with 5% BSA (Roche, Basel, Switzerland), overnight incubation with the designated primary antibodies at 4°C, and three washes with PBS/0.1% Tween 20 (Servicebio), the cells were incubated for 1 hour at room temperature with the corresponding secondary antibodies. A Zeiss Axio Imager M2 fluorescence microscope (Zeiss, Oberkochen, Germany) was used to obtain fluorescence images. Anti-vimentin (#BM4029; Boster, Wuhan, China) was used as the primary antibody.

### Oil Red O Staining

GO-OFs in 6-well plates were induced using adipogenic differentiation medium (Oricell, Guangzhou, China). After the adipocytes formed clusters (between 12 and 18 days), they were concurrently treated with simvastatin or DMSO. After 72 hours of incubation, Oil Red O staining was performed. The cells were washed twice with PBS, fixed with 10% formalin for 30 minutes, stained with Oil Red O for 1 hour, and then rinsed twice with water. The Oil Red O staining procedure was followed. Subsequently, 300 µL pure isopropanol (Xilong, Shanghai, China) was added to each well for 5 minutes to extract lipids. The extracted solution was then diluted by a factor of 20, and the absorbance was measured at a wavelength of 470 nm using a microplate detector (Tecan, Männedorf, Switzerland). Additionally, the cells were visually captured using a phase-contrast microscope at 50×, 100×, and 200× magnification.

### Cell Proliferation and Viability Assay

After overnight incubation, the cells (3 × 10^3^) were seeded in 96-well plates and treated for 24, 48, or 72 hours with the following reagents: erastin (Selleckchem, Houston, Texas, USA), ferrostatin 1 (Fer-1; Selleckchem), simvastatin (MCE, Princeton, New Jersey, USA), Z-Val-Ala-Asp (OMe)–fluoromethylketone (Z-VAD-FMK; Selleckchem), 3-methyladenine (3-MA; Selleckchem), or DMSO (Sigma, St. Louis, MO, USA). Cell viability was assessed 24 to 72 hours posttreatment using a Cell Counting Kit-8 (CCK-8; Dojindo, Kumamoto, Japan), and the absorbance at 450 nm was measured to evaluate cell proliferation in each well. Each experiment was repeated at least three times, and relative cell viability was determined by comparison to that of the control or GO group that was treated with DMSO. After measuring the half-maximal inhibitory concentration of simvastatin on GO-OFs using CCK-8, GO-OFs were treated with 60 µM simvastatin for 72 hours for subsequent experiments.

### Lipid Peroxidation Assay

Lipid peroxidation was assessed using BODIPY 581/591 C11 (MCE). In summary, cells were extracted and exposed to BODIPY 581/591 C11 (2 µM) for 30 minutes. The samples were then examined using a flow cytometer (Cytek, Fremont, California, USA).

### RNA Extraction and Real-Time PCR

Total RNA was extracted using TRIzol reagent (Invitrogen, Carlsbad, CA, USA), and a cDNA synthesis kit (MCE) was used to reverse transcribe 2 µg total RNA. The ABI Quant Studio 5 system (Thermo Fisher, Waltham, Massachusetts, USA) from Applied Biosystems was used for quantitative PCR, and real-time PCR master mix and SYBR Green dye (TaKaRa, Kyoto, Japan) were used. GAPDH served as a control, and the reactions were performed in triplicate. The cycle threshold (Ct) was used to calculate the relative mRNA expression levels, which were then adjusted to GAPDH levels using the 2^–^^ΔΔCt^ method. Primers (Sangon Biotech, Shanghai, China) that were used are listed in Table 3.

**Table 3.** Primer Sequences for Lipid Metabolism Genes

Primer	Sequence
GAPDH forward	CTGGGCTACACTGAGCACC
GAPDH reverse	AAGTGGTCGTTGAGGGCAATG
ACSL4 forward	GACTGGCAAGAAGGCGGTTATAC
ACSL4 reverse	GGAGATGTTCTGTCCACCAATTACG
HMGCR forward	ACCTTTCCAGAGCAAGCACATTAG
HMGCR reverse	CCAACTCCAATCACAAGACATTCAAC
ACC forward	TCTCCTCCAACCTCAACCACTATG
ACC reverse	ATTCCGCCCATCCGCTGAC
FASN forward	CAGCGGCAAGCGTGTGATG
FASN reverse	CCTCCTCCAGCGTCCAGTTG
SREBF1 forward	ACTGGTCGTAGATGCGGAGAAG
SREBF1 reverse	TGTCATTGATGGAGGAGCGGTAG
CEBPα forward	CTGGACGGCAGGCTGGAG
CEBPα reverse	GGCGGCGGCTGGTAAGG
PPARγ forward	TCCGTGGATCTCTCCGTAATGG
PPARγ reverse	TTCTTGTGAATGGAATGTCTTCGTAATG

ACC, acetyl-CoA carboxylase; ACSL4, Acyl-CoA synthetase long-chain family member 4; CEBPα, CCAAT/enhancer binding protein alpha; FASN, fatty acid synthase; GAPDH, glyceraldehyde-3-phosphate dehydrogenase; PPARγ, peroxisome proliferator-activated receptor gamma; SREBF1, sterol regulatory element binding transcription factor 1.

### Western Blot Analysis

After being lysed with RIPA buffer (Solarbio, Beijing, China), the cell lysates were separated by SDS-PAGE, blotted onto membranes, blocked with 5% BSA for 1 hour, and immunoblotted with the following primary antibodies at 4°C overnight: actin (#ab8226, 1/1000 dilution; Abcam, Cambridge, UK), ACLS4 (#sc271800, 1/500 dilution; Santa, Santa Cruz, California, USA), and HMGCR (#TD6518, 1/1000 dilution; Abmart, Shanghai, China). Finally, the membranes were incubated with secondary antibodies conjugated to horseradish peroxidase (1/2500 dilution; ZSBG Bio, Beijing, China) and visualized by imaging systems.

### Transmission Electron Microscopy

Centrifuged treated OFs were embedded in an electron microscope fixative (Hitachi, Tokyo, Japan). The fixed cell slices were embedded in Epok 812 after being postfixed with 2% OsO_4_ and dried in ethanol. An ultramicrotome was used to cut ultrathin sections, which were then stained with lead citrate and uranyl acetate and analyzed under an electron microscope.

### Measurement of Intracellular ROS

ROS were examined by assessing the intracellular peroxide-dependent oxidation of DCFH-DA (Beyotime), resulting in the formation of the fluorescent molecule 2′,7′-dichlorofluorescein (DCF). The cells were placed onto 6-well plates at a density of 12 × 10^4^ cells per well and incubated for 24 hours. Following two washes with PBS, fresh medium containing 60 µM simvastatin was added to the cells, which were then incubated for 24 hours. Subsequently, a solution containing 20 µM DCFH-DA was added to the cells, which were then subjected to a 30-minute incubation period at 37°C. The cells were rinsed twice with PBS, followed by the addition of 400 µL PBS to each well. The fluorescence intensity was then measured using a fluorescence microscope (Olympus, Tokyo, Japan).

### Measurement of Intracellular Iron

To determine the levels of intracellular ferrous ions, a commercial kit (Elabscience, Wuhan, China) was utilized. Before harvesting, cells were washed with PBS and subsequently divided for cell enumeration and further analysis. A buffer solution was added at a ratio of 200 μL per 1 × 10^6^ cells to induce cell lysis. This lysis process occurred on ice for 30 minutes, with intermittent vortexing every 10 minutes. Following centrifugation at 15,000 *g* for 10 minutes at 4°C, the supernatant was collected and combined with 80 μL of a chromogenic agent. The mixture was then incubated in the dark at 37°C for 90 minutes. Absorbance readings were taken at 593 nm for all samples. The total concentration of intracellular ferrous ions was derived from a standard curve and adjusted based on cell counts.

### Statistics

GraphPad Prism 9.3.1 (GraphPad Software, La Jolla, CA, USA) was used for data analysis. All experiments were performed at least three times using a minimum of three replicates per condition in each experiment. The data were presented as the mean ± SD. The χ^2^ test was used to assess the differences in gender composition between the GO and control groups. For the comparison between the GO and control groups, as well as between untreated and treated cultures, unpaired *t*-tests were employed for all other statistical metrics, encompassing age, mRNA expression levels, OD values, relative CCK-8 intensity, lipid peroxidation levels, and the concentration of ferrous ions. *P* < 0.05 was considered to indicate statistical significance.

## Results

### Simvastatin Inhibits the Adipogenesis of GO-OFs

**Figure 1.**
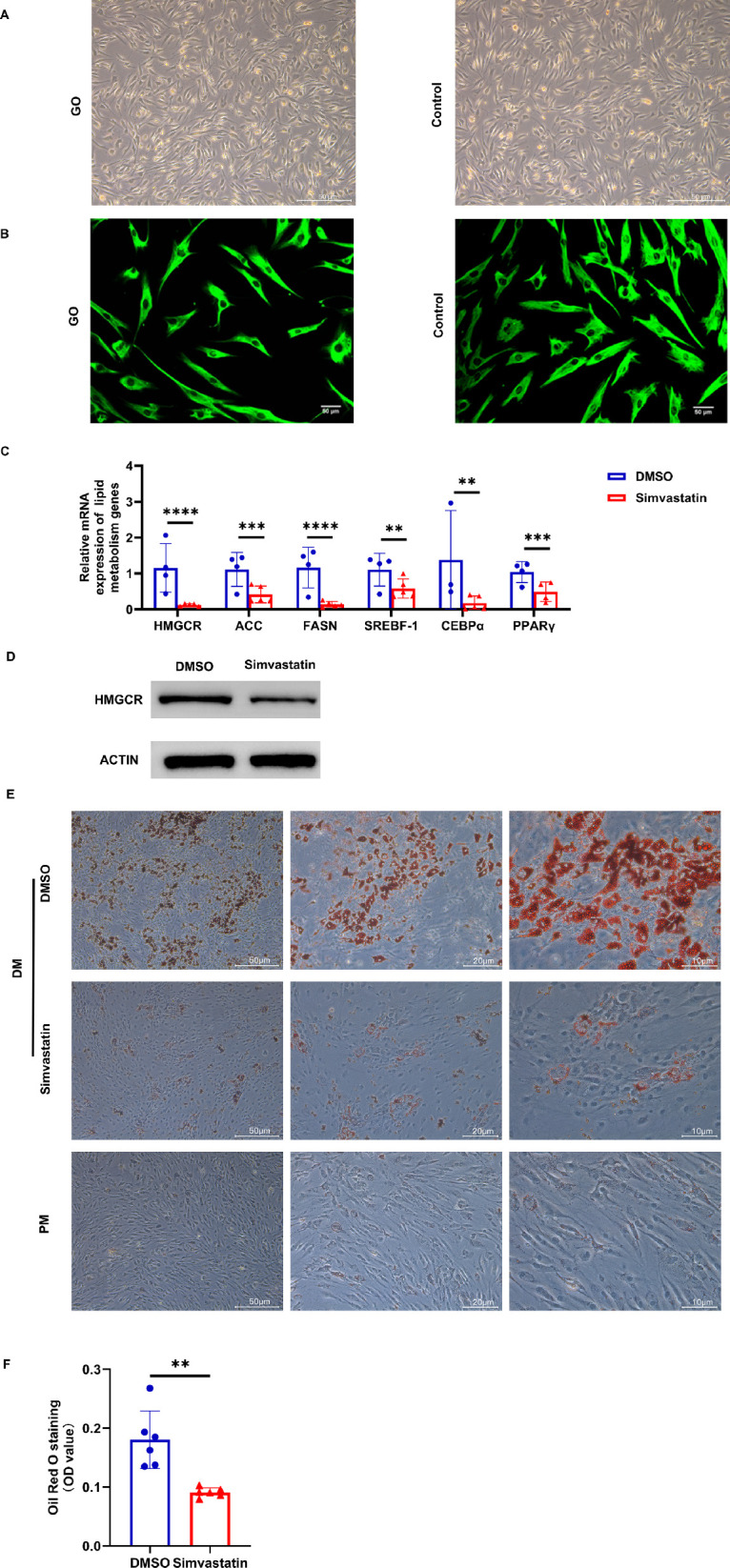
Isolation and identification of OFs. (**A**) The morphology of primary cultured GO-OFs and control OFs is shown in bright-field images. *Scale bar*: 50 µm. (**B**) Immunofluorescence staining for vimentin was positive in both the GO-OFs and control OFs. *Scale bar*: 50 µm. (**C**) The relative mRNA expression of adipogenic marker genes (such as PPARγ, CEBPα, ACC, FASN, SREBF-1, and HMGCR) was analyzed by RT-qPCR following 60 µM simvastatin administration for 72 hours. *n* = 3–5. (**D**) After 60 µM simvastatin administration for 72 hours, the protein levels of HMGCR were assessed by Western blot analysis. (**E**) GO-OFs were taken as they were cultured in differentiation medium (DM) and proliferation medium (PM); after the adipocytes formed clusters (between 12 and 18 days), they were concurrently treated with 60 µM simvastatin or DMSO. After 72 hours of incubation, Oil Red O staining was performed. *Scale bar*: 50 µm, 20 µm, 10 µm. (**F**) Quantification of Oil Red O staining in differentiated GO-OFs. The graph displays the relative quantification based on the absorbance at 470 nm. *n* = 6. The data are shown as the mean ± SD. **P < 0.01, ***P < 0.001, ****P < 0.0001.

The spindle- or star-shaped morphology of the orbital fibroblasts was observed by microscopic inspection (Fig. 1A). Vimentin-containing orbital fibroblast cytoplasm was positive and stained green according to immunofluorescence staining (Fig. 1B). Subsequently, we analyzed the mRNA levels of genes involved in the development of adipogenesis and lipogenesis in the GO-OFs treated with simvastatin. The results showed that simvastatin significantly reduced the expression of adipogenic marker genes (PPARγ, CEBPα, ACC, FASN, SREBF-1, and HMGCR) (Fig. 1C). Simvastatin also led to a decrease in HMGCR protein expression (Fig. 1D). The differentiation level of GO-OFs treated with simvastatin or DMSO was assessed by Oil Red O staining. After inducing adipogenesis, simvastatin and DMSO were added to GO-OFs, respectively. Microscopic images showed that the quantity and clustering of mature adipocytes in the simvastatin-treated group were significantly reduced (Fig. 1E). This finding was also confirmed following the extraction process using isopropanol (Fig. 1F). These findings indicate that simvastatin inhibits lipogenesis in GO-OFs.

### GO-OFs Are Resistant to Erastin-Induced Ferroptosis

**Figure 2.**
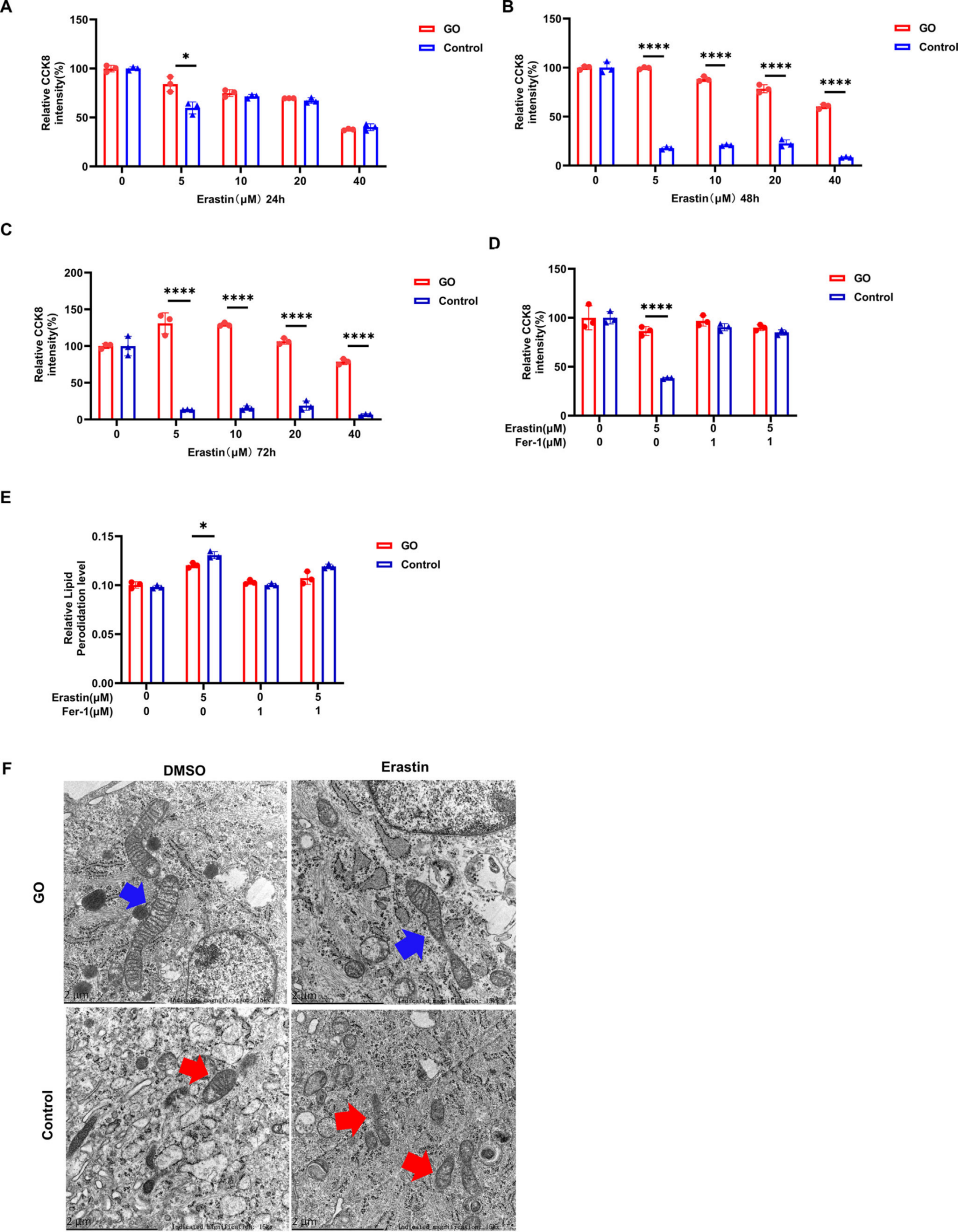
GO-OFs exhibit increased resistance to erastin-induced ferroptosis. (**A–C**) Comparison of cell viability between the GO-treated and control OFs after ferroptosis induction. OFs were seeded at 3000 cells per well in 96-well plates and subjected to a CCK-8 viability assay after incubation with different concentrations of erastin in the GO and control groups for 24, 48, and 72 hours. The percentage representation of the relative CCK-8 intensity is achieved by normalizing it against the mean OD value attained from the wells subjected to DMSO treatment. *n* = 3. (**D**) The effects of treatment with DMSO, erastin, Fer-1, or erastin + Fer-1 on the viability of GO-OFs and control-OFs for 48 hours, as determined by a CCK-8 viability assay. *n* = 3. (**E**) Comparison of lipid peroxidation between the GO-OFs and control-OFs after ferroptosis induction (5 µM erastin and/or 1 µM Fer-1 for 48 hours). Lipid peroxide levels in the OFs were measured by flow cytometry, and relative lipid peroxide levels are shown on the y-axis. The levels of lipid peroxides exhibited a considerable disparity between the erastin-treated GO group and the control group. *n* = 3. (**F**) The mitochondrial morphology of the OFs treated with DMSO and 5 µM erastin for 48 hours is shown via transmission electron microscopy (*blue arrow*: mitochondria did not become significantly smaller, with obvious cristae; *red arrow*: mitochondria decreased and shrank, with cristae reduced or even eliminated). *Scale bar*: 2 µm. The data are shown as the mean ± SD. *P < 0.05, ****P < 0.0001.

GO-OFs and control-OFs were treated with 5, 10, 20, or 40 µM erastin for 0 to 72 hours. When GO-OFs were treated with 5 to 40 µM erastin one by one, it was found that the viability of GO-OFs decreased with the increase in concentration (Figs. 2A–C). The survival rate of the control-OFs decreased more with time. The concentration of erastin that produced the greatest difference in cell activity between the GO group and the control group was 5 µM. Therefore, 5 µM erastin was ultimately chosen for subsequent experiments. After the addition of ferroptosis inhibitors (Fer-1), the survival rate of the OFs in both groups was restored (Fig. 2D). After erastin treatment, the lipid peroxidation level of the OFs in both groups increased compared to that in the DMSO treatment group. However, the increase in lipid peroxidation was lower in the GO-OFs than in the control-OFs (Fig. 2E). Transmission electron microscopy (TEM) showed that the mitochondria in the control-OFs decreased in size and shrank, and the cristae decreased in size or even disappeared; however, the mitochondria in the GO-OFs were relatively intact, with obvious cristae and no obvious changes in size (Fig. 2F). Taken together, these data suggest that GO-OFs are resistant to erastin-induced ferroptosis.

### Fer-1 Reverses the Cell Death of GO-OFs Induced by Simvastatin

**Figure 3.**
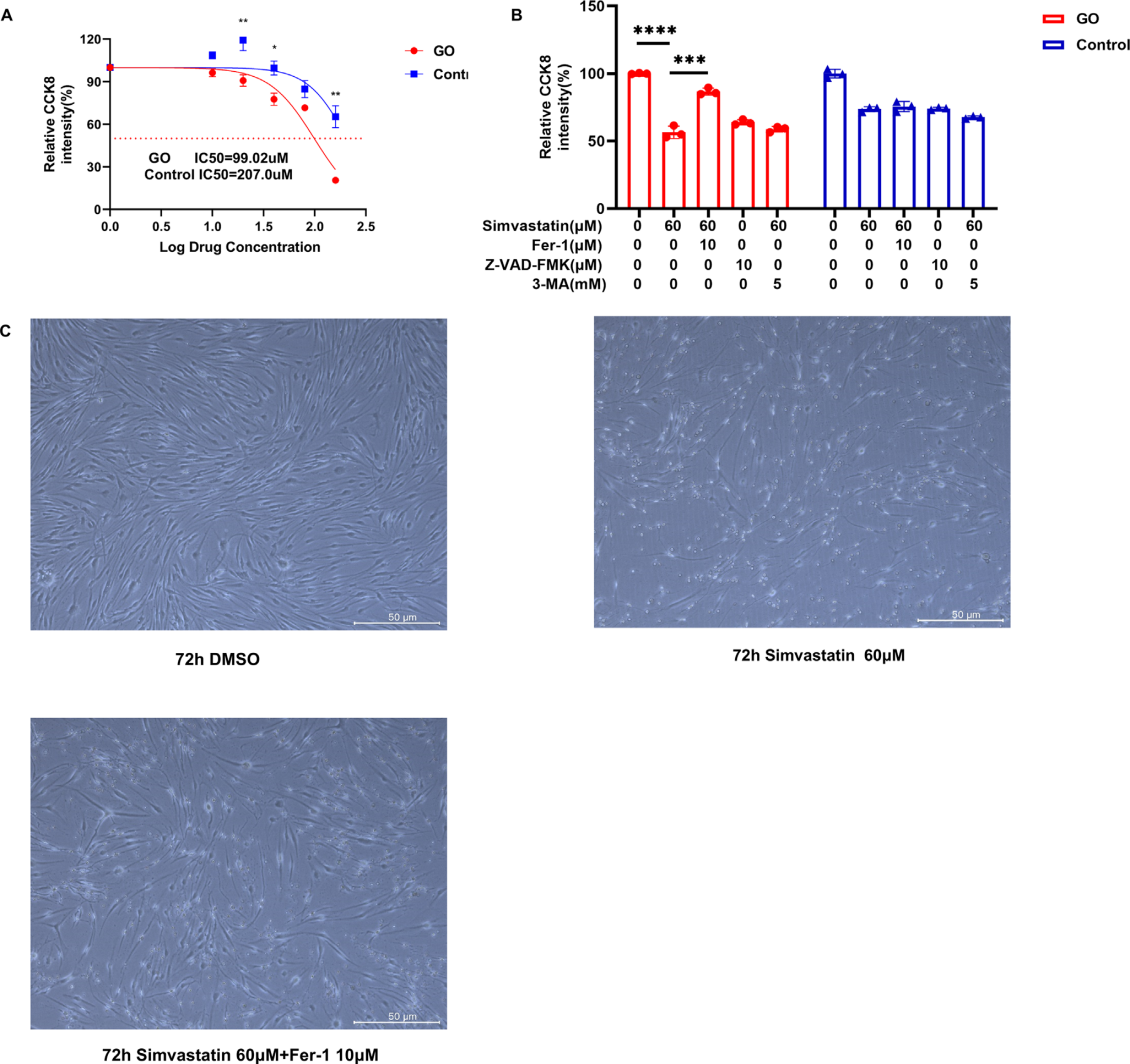
Fer-1 reversed the cell death of GO-OFs induced by simvastatin. (**A**) The effects of 72 hours of treatment with simvastatin (0–160 µM) on the viability of the GO-treated and control OFs were measured by a CCK-8 cell viability assay. *n* = 3. (**B**) GO-OFs were treated with 60 µM simvastatin for 72 hours with or without 10 µM Fer-1/10 µM Z-VAD-FMK/5 mM 3-MA. CCK-8 assays were performed to assess cell growth. *n* = 3. (**C**) After the addition of various treatments, specific variations in both cell density and morphology were observed by microscopy. *Scale bar*: 50 µm. The data are shown as the mean ± SD. *P < 0.05, **P < 0.01,***P < 0.001, ****P < 0.0001.

With increasing concentrations of simvastatin, the survival rate of OFs decreased. The half maximal inhibitory concentration (IC50) of the GO-OFs was lower than that of the control-OFs (Fig. 3A). The growth of the GO-OFs was notably inhibited by treatment with 60 µM simvastatin for 72 hours (*P* < 0.05). Moreover, these inhibitory effects were significantly reversed after the addition of the ferroptosis inhibitor Fer-1 to the GO-OFs (Fig. 3B), but the apoptotic inhibitor Z-VAD-FMK and the autophagy inhibitor 3-MA were unable to reverse the cell death caused by simvastatin in GO-OFs (Fig. 3B). The morphology of the GO-OFs changed from spindle-shaped to round after the addition of simvastatin. Furthermore, the morphologic changes observed in the GO-OFs were reversed after the addition of Fer-1 (Fig. 3C). These findings indicate that simvastatin may trigger ferroptosis in GO-OFs.

### Simvastatin Induces Ferroptosis in GO-OFs

**Figure 4.**
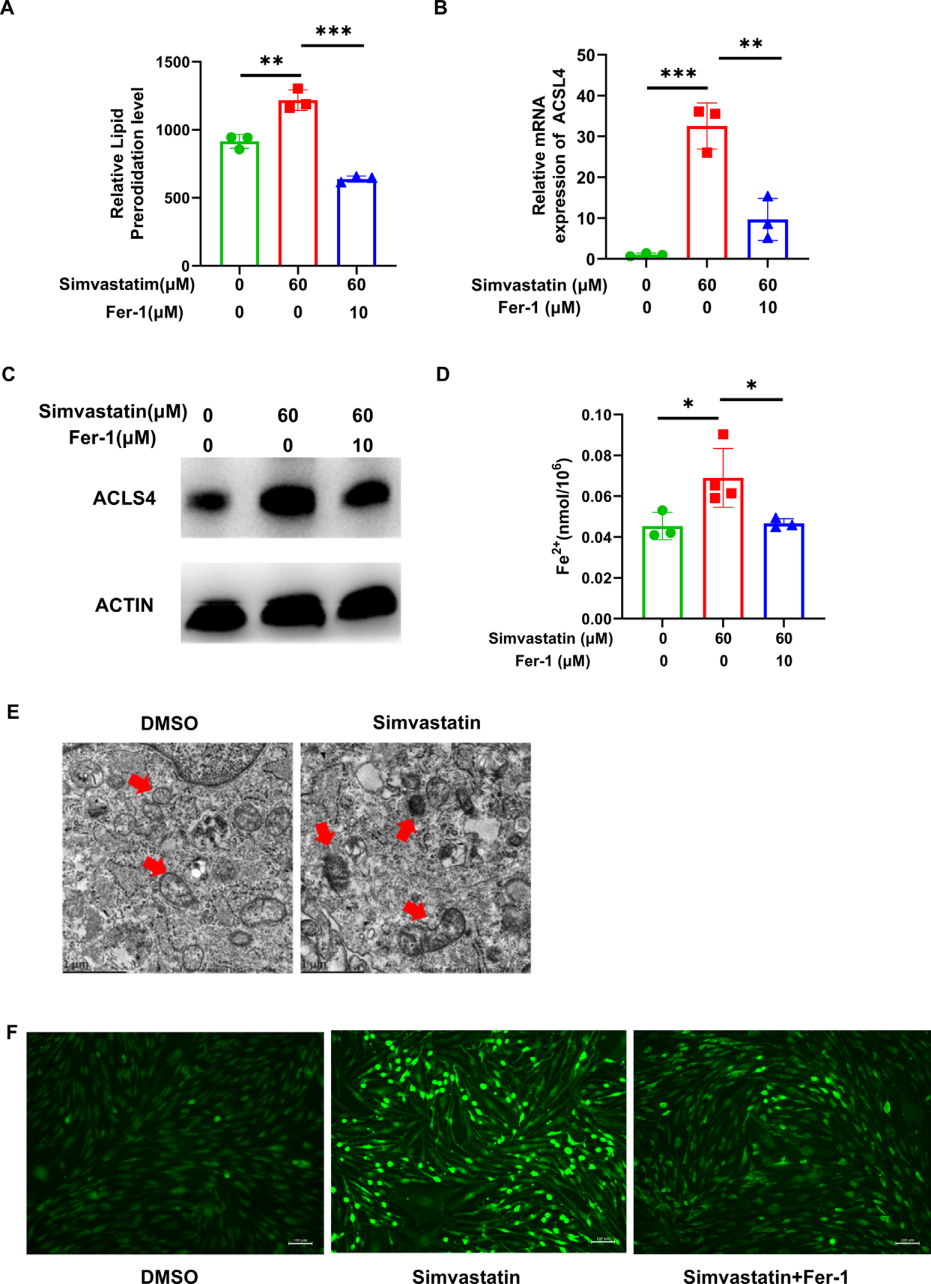
Simvastatin-induced ferroptosis in GO-OFs. (**A**) Relative lipid peroxidation level of GO-OFs treated with 60 µM simvastatin for 72 hours in the absence or presence of 10 µM Fer-1, as determined by flow cytometry. *n* = 3. (**B**) After 60 µM simvastatin for 72 hours with or without 10 µM Fer-1, the mRNA expression of ACLS4 was measured by RT-qPCR. *n* = 3. (**C**) After 60 µM simvastatin for 72 hours with or without 10 µM Fer-1, the protein expression of ACLS4 in GO-OFs was assessed by Western blot analysis. (**D**) The concentration of Fe^2+^ was assessed following a 72-hour treatment with 60 µM simvastatin, either alone or in combination with 10 µM Fer-1. *n* = 3–4. (**E**) After treatment with 60 µM simvastatin for 72 hours, the GO-OFs had ruptured outer membranes, condensed densities in the mitochondrial membrane, disappearance of mitochondrial cristae, and rounding of the mitochondria, as shown by TEM (*red arrows*). *Scale bar*: 1 µm. (**F**) Representative photomicrographs of the GO-OFs stained with DEFH fluorescent dye after exposure to 60 µM simvastatin for 72 hours with or without 10 µM Fer-1. *Scale bar*: 100 µm. The data are shown as the mean ± SD. *P < 0.05, **P < 0.01, ***P < 0.001.

C11-BODIPY was utilized to assess lipid peroxidation in GO-OFs. Flow cytometry revealed that the GO-OFs treated with simvastatin exhibited significantly greater lipid peroxidation than the controls. As expected, Fer-1 inhibited simvastatin-induced lipid peroxidation (Fig. 4A). Furthermore, simvastatin increased the expression of ferroptosis markers (acyl-CoA synthetase long-chain family member 4, ACLS4), and this effect was inhibited by Fer-1 (Figs. 4B, 4C). Similarly, an increase in ferrous ion concentration was observed in GO-OFs after simvastatin treatment (Fig. 4D). When simvastatin was administered concurrently with Fer-1, the concentration decreased compared to the simvastatin group (Fig. 4D). After treatment with simvastatin, significant changes in the mitochondrial ultrastructure of the GO-OFs, including ruptured mitochondrial outer membranes, condensed mitochondrial membrane densities, missing mitochondrial cristae, and mitochondria rounding, were observed via TEM (Fig. 4E, red arrows). The fluorescence intensity of ROS in the simvastatin group was greater than that in the DMSO group when DCFH-DA was added to the cell culture medium (Fig. 4F). The intensity of the fluorescence diminished subsequent to the concurrent introduction of simvastatin and Fer-1 (Fig. 4F). Overall, simvastatin can trigger ferroptosis in GO-OFs, and this effect can be reversed by Fer-1.

## Discussion

Our study revealed that GO-OFs exhibited resistance to ferroptosis. After the administration of simvastatin, decreased adipogenesis and characteristics linked to ferroptosis, such as changes in the structure of the mitochondria, increased ROS, increased lipid peroxidation, and increased mRNA and protein expression of ACLS4, were observed for the GO-OFs. These results indicated that simvastatin inhibits the growth of GO-OFs by inducing ferroptosis.

GO is a multifactorial autoimmune disease characterized by inflammation and remodeling of orbital tissues that affects approximately 25% to 30% of individuals newly diagnosed with hyperthyroidism due to Graves’ disease. The hyperplasia of retrobulbar orbital adipose tissue plays a crucial role in the pathology of GO. OFs are considered central target cells in the pathogenesis of GO, which is involved in the physiologic processes of orbital inflammatory infiltration, oxidative stress, adipogenesis, and fibrosis. Recent evidence has shown that statins have a protective effect against the progression of GO in individuals with Graves’ disease, and the use of nonstatin cholesterol-lowering drugs was not associated with a reduced risk of GO in an American study. The protective effect of statins has been suggested to be related to other mechanisms.^7^ Our study provided in vitro evidence that simvastatin inhibits the growth and adipogenesis of GO-OFs by inducing ferroptosis in GO, which may constitute a novel mechanism by which statins protect individuals with GO.

Ferroptosis is considered a type of programmed cell death that involves mechanisms other than apoptosis, necrosis, and autophagy. Emerging evidence suggests that ferroptosis is involved in the development of cancer, cardiovascular disease, neurodegenerative disease, and autoimmune disease. Statins (such as simvastatin) are a class of drugs used to lower blood cholesterol levels through the inhibition of HMGCR. The suppression of HMGCR by statins leads to decreased expression of GPX4 and CoQ10, which in turn results in lipid ROS production and the induction of ferroptosis. Lipid peroxidation is a hallmark of ferroptosis.^26^ ACSL4 is an essential target in both ferroptosis and fatty acid metabolism.^27^ Prior research has shown that GO-OFs exhibit resistance to ferroptosis and that glycolysis can expedite the progression of ferroptosis.^24^ Our study also confirmed that GO-OFs are resistant to ferroptosis (Fig. 2). Following the administration of erastin to promote ferroptosis in GO-OFs and control OFs, we observed that the control OFs exhibited increased production of lipid peroxides. Furthermore, when we added simvastatin to GO-OFs, we found that simvastatin contributed to an increase in lipid peroxidation, while Fer-1 counteracted the above effects. These results indicated that simvastatin induces ferroptosis in GO-OFs.

We further explored the mechanism by which ferroptosis is induced by simvastatin. Increased expression of ACLS4 was observed in the GO-OFs treated with simvastatin (Figs. 4B, 4C); moreover, reduced expression of HMGCR was observed in our experiments (Figs. 1C, 1D). ACSL4 is a key participant in lipid peroxidation and is necessary for ferroptosis.^28^^–^^31^ The phosphorylation of ACSL4 is a critical factor in the amplification of excess lipid peroxidation, leading to the acceleration of ferroptosis.^32^ Overexpression of ACSL4 is thought to induce ferroptosis, as it is regarded as a crucial regulator of ferroptosis.^30^ Research has shown that ACSL4 affects the vulnerability of fibroblasts to ferroptosis.^33^ ACSL4 exhibits a preference for polyunsaturated fatty acids (PUFAs), which are recognized as one of the favored substrates for ferroptosis.^34^^,^^35^ ACSL4 links PUFAs to CoA, producing fatty acyl-CoA esters.^36^ Acetyl-CoA is a specific type of fatty acyl-CoA that can be transformed into HMG-CoA by the catalytic action of HMGCR. As this enzyme is a rate-limiting enzyme in the mevalonate pathway, simvastatin can inhibit this process. Based on our experimental results, we speculate that simvastatin induces ferroptosis in GO by upregulating the expression of ACLS4 and reducing the expression of HMGCR in GO-OFs.

Our study has several limitations. The tissues included in this study were sourced from individuals whose clinical activity score was less than 3, and thus, these samples may not fully capture the inflammatory condition because orbital surgery is rarely advised for individuals with active GO except for those with an urgent need. Furthermore, similar to other in vitro investigations, our study focused solely on OFs without considering the interplay between immune-active cells and OFs or the potential impact of the microenvironment. In addition, the bioavailability of simvastatin is low, and it is mainly bound to plasma proteins. The actual exposure of cells in the body to free simvastatin may be much lower than the total plasma concentration. Therefore, in the absence of in vivo studies, high concentrations of simvastatin may limit the clinical significance of the results. Further studies are needed. For example, the use of nanomaterials to encapsulate simvastatin for local administration (e.g., via periocular injection) could be considered to optimize drug delivery and minimize systemic effects.

In conclusion, we revealed that simvastatin has a protective effect on GO by regulating the ferroptosis of GO-OFs. As a safe and readily available medication, simvastatin may have the potential to be used as a complementary treatment for GO. Further research is required to investigate the precise molecular mechanisms involved.
